# Roots drive oligogalacturonide‐induced systemic immunity in tomato

**DOI:** 10.1111/pce.13917

**Published:** 2020-11-03

**Authors:** Jordi Gamir, Zhivko Minchev, Estefanía Berrio, Juan M. García, Giulia De Lorenzo, Maria J. Pozo

**Affiliations:** ^1^ Department of Soil Microbiology and Symbiotic Systems Estación Experimental del Zaidín (CSIC) Granada Spain; ^2^ Present address: Metabolic Integration and Cell Signaling Group, Plant Physiology Section, Unidad Asociada a la EEZ‐CSIC, Dept Ciencias Agrarias y del Medio Natural, Universitat Jaume I Castellón Spain; ^3^ Dipartimento di Biologia e Biotecnologie C. Darwin Sapienza Università di Roma Rome Italy

**Keywords:** alkaloids, *Botrytis cinerea*, DAMPs, flavonoids, metabolomics, phytohormones, plant defences, systemic resistance

## Abstract

Oligogalacturonides (OGs) are fragments of pectin released from the plant cell wall during insect or pathogen attack. They can be perceived by the plant as damage signals, triggering local and systemic defence responses. Here, we analyse the dynamics of local and systemic responses to OG perception in tomato roots or shoots, exploring their impact across the plant and their relevance in pathogen resistance. Targeted and untargeted metabolomics and gene expression analysis in plants treated with purified OGs revealed that local responses were transient, while distal responses were stronger and more sustained. Remarkably, changes were more conspicuous in roots, even upon foliar application of the OGs. The treatments differentially activated the synthesis of defence‐related hormones and secondary metabolites including flavonoids, alkaloids and lignans, some of them exclusively synthetized in roots. Finally, the biological relevance of the systemic defence responses activated upon OG perception was confirmed, as the treatment induced systemic resistance to *Botrytis cinerea*. Overall, this study shows the differential regulation of tomato defences upon OGs perception in roots and shoots and reveals the key role of roots in the coordination of the plant responses to damage sensing.

## Abstract 2

Oligogalacturonide perception in tomato roots or shoots triggers differential transient local responses, but also a myriad of systemic responses at the metabolic, transcriptomic and enzymatic level leading to pathogen resistance. The results highlight the key role of roots in the regulation of these systemic responses.

## 1 INTRODUCTION

Plants respond to invading organisms triggering fast signalling processes leading to the activation of diverse defence mechanisms. They have adapted their immune system to rely on an early molecular recognition of the potential aggressor, crucial for an efficient defence reaction (Jones & Dangl, 2006). Immune responses are controlled by pattern recognition receptors, and defence signalling starts with the perception of conserved molecules associated to the damaging organism, such as pathogen (or microbe)‐associated molecular patterns (PAMPs or MAMPs). Moreover, they can also recognize self‐molecules associated to damage, the so‐called damage‐associated molecular patterns (DAMPs) (Zipfel & Oldroyd, 2017).

DAMPs are damaged‐self molecules released from host tissue disruption that act as endogenous danger signals in both animals and plants (Heil & Land, 2014). DAMPs comprise a mixture of molecules from diverse origin such as extracellular nucleotides (eATP, eDNA and eNAD[P]), inducible proteins and fragments of the cell wall (Heil & Land, 2014; Li, Wang, & Mou, 2020). In plants, DAMPs are released from disintegrated cells and are sensed by the pattern recognition receptors of adjacent cells. After the recognition, plants go into an “alarm state” activating signalling cascades and triggering defence responses not only locally, at the damaged tissue, but also in distal tissues that will then be prepared to respond more efficiently to a potential upcoming aggression (Orozco‐Cardenas & Ryan, 1999). Local responses to DAMPs involve the generation of H_2_O_2_, MAPKs activation, increased flux of calcium, production of phenylpropanoids and hypomethylation in CpG sites (Barbero, Guglielmotto, Capuzzo, & Maffei, 2016; Duran‐Flores & Heil, 2017; Pétriacq, Ton, Patrit, Tcherkez, & Gakière, 2016; Vega‐Muñoz, Feregrino‐Pérez, Torres‐Pacheco, & Guevara‐González, 2018). Damage perception also involves cell‐to‐cell communication to prime distal parts of the plant. Consequently, plants activate a myriad of mobile signals that transmit the alarm state and activate defence responses over long distances. It has been reported that jasmonic acid (JA) signalling mediates some of the systemic responses in tomato plants after DAMPs perception (Sun, Jiang, & Li, 2011). Generation of hydrogen peroxide, accumulation of proteinase inhibitors and other defence‐related proteins are produced in distal leaves upon wounding or application of the peptidic, wound‐related hormone systemin in tomato (Orozco‐Cardenas & Ryan, 1999; Sun et al., 2011).

Oligogalacturonides (OGs) are among the best characterized plant DAMPs. They are pectin fragments hydrolysed from the cell wall that act as danger signals, triggering a signalling cascade that activates plant immunity (De Lorenzo, Ferrari, Cervone, & Okun, 2018; Ferrari et al., 2013; Savatin, Gramegna, Modesti, & Cervone, 2014). OGs are oligomers of α‐1,4‐galacturonic acid that are released to the extracellular cell space through the action of polygalacturonases, usually generated during pathogens or insects attack (Benedetti et al., 2015). Exogenous application of OGs induces defence responses in plants when they have a degree of polymerization between 10 and 15 and they have acquired an egg‐box conformational state dependent on calcium and sodium (Benedetti et al., 2015; Cabrera, Boland, Messiaen, Cambier, & Van Cutsem, 2008). Short oligomers have been also shown to trigger plant defences, although to a lesser extent than long OGs (Davidsson et al., 2017).

It has been demonstrated that OGs perception stimulates antioxidant systems in plants (Camejo et al., 2012) and the biosynthesis of different antimicrobial enzymes through responses regulated by the main defence related phytohormones: JA, salicylic acid (SA) and ethylene (ET) (Bishop, Pearce, Bryant, & Ryan, 1984; Denoux et al., 2008; Doares, Syrovets, Weiler, & Ryan, 1995; Ferrari et al., 2007; Gravino, Savatin, Macone, & De Lorenzo, 2015). These hormonal signalling pathways play a key regulatory function in the interaction of plants with potential aggressors as pathogens and herbivores (Pieterse et al., 2014). Therefore, the modulation of these pathways by OGs would likely have a relevant impact in these biotic interactions.

The ability of OGs to induce defence responses in plants stimulated the scientific community to study the potential of OGs for plant protection. In grape, pre‐incubation of excised leaves with OGs leads to protection against the necrotrophic pathogen *Botrytis cinerea* (Aziz, Heyraud, & Lambert, 2004), and protection was also achieved in Arabidopsis by spray‐application of OGs (Ferrari et al., 2007; Galletti et al., 2008). Moreover, in‐vivo production of bioactive OGs oligomers in Arabidopsis boosts plant defences and induces resistance to necrotrophic and biotrophic pathogens (Benedetti et al., 2015). Some research efforts have been devoted to analyse the plant responses to OGs that mediate this locally induced enhanced resistance. In Arabidopsis, OG‐induced resistance against *B. cinerea* does not require JA and SA signalling, nor the oxidative burst generated in plants by OG perception (Aziz et al., 2004; Ferrari et al., 2007; Galletti et al., 2008; Galletti, Ferrari, & De Lorenzo, 2011; Gravino et al., 2015). Instead, it requires a functional *PAD3*, which encodes the last step of camalexin biosynthesis (Ferrari et al., 2007. Based on previous evidences, formulations combining OGs with chitosan oligomers are already available for plant protection against pathogens (van Aubel, Cambier, Dieu, & Van Cutsem, 2016).

Little is known about the responses induced by OGs at the systemic level, despite the well‐established relevance of systemic defence responses in plants (Hilleary & Gilroy, 2018). The first observations of the function of OGs as an elicitor of systemic responses were obtained in tomato (Bishop, Makus, Pearce, & Ryan, 1981; Reymond, Grünberger, Paul, Müller, & Farmer, 1995; Simpson, Ashford, Harvey, & Bowles, 1998; Thain, 1995). However, the induction of systemic resistance to pathogens upon OG treatment has been so far reported only in Arabidopsis (Ferrari et al., 2007), although the molecular mechanisms behind this response are unexplored. Tomato was one of the model plants for the pioneer studies addressing systemic wound responses (Birkenmeier & Ryan, 1998; O'Donnell et al., 1996; Schilmiller & Howe, 2005) and tomato defence responses against *B. cinerea* are known to involve the wound related hormones JA, SA, ET and abscisic acid (ABA) (Achuo, Audenaert, Meziane, & Höfte, 2002; Asselbergh et al., 2007; Curvers et al., 2010; Díaz, ten Have, & van Kan, 2002; El Oirdi et al., 2011). Hence, an important question is how tomato plants respond not only locally but also systemically to OGs recognition and if these responses are able to trigger induced resistance (IR) against pathogens.

In this study we examined how tomato plants coordinate local and systemic responses to OG perception in different organs. In addition, we addressed the biological relevance of these responses by testing their efficacy in enhancing plant resistance against *B. cinerea*, a common and polyphagous necrotrophic pathogen. We show that changes in hormone levels induced by OGs are fast and transient at the local level and more sustained at the systemic level, and notably, that OGs have a stronger impact in roots than in leaves, regardless of the application site. Untargeted metabolomic analysis highlights the differential response to OGs in local and systemic tissues, supporting the notion of a precise fine‐tuning of plant defences in response to this class of DAMPs, and uncovers the major pathways targeted by OG signalling. Finally, we show that root or leaf treatment with OGs induces systemic resistance against *B. cinerea* in tomato plants. The results highlight the differences among local and systemic responses and their dependence on the site of signal perception.

## 2 MATERIAL AND METHODS

### 2.1 Plant material and growth conditions

Tomato seeds (*Solanum lycopersicum*, cv Castelmart) were surface sterilized in 4% sodium hypochlorite, rinsed thoroughly with sterile water and germinated for 3 days on moistened filter paper at 25°C in darkness. Subsequently, seedlings were transferred into 3 L plastic containers and grown hydroponically with water during the first week and with 0.5x Long Ashton nutrient solution (Hewitt, 1966) until the end of the experiment. The nutrient solution was replaced by fresh solution once a week.

### 2.2 Oligogalacturonide treatments

Oligogalacturonides (DP 10‐15) were prepared as previously described (Benedetti et al., 2017): A PGA solution (2% [w/v; Alfa Aesar]) was incubated with endo‐polygalacturonase II (0.1 RGU/ml), purified from *Aspergillus niger* Pectinase (Sigma), for 180 min at 30°C in a water bath under gentle shaking. The digest was boiled for 10 min in a water bath to inactivate the enzyme and cooled at 4°C on ice. Oligogalacturonides were precipitated by diluting the solution with cold 50 mM sodium acetate and ice‐cold ethanol to a final concentration of 0.5% (w/v) PGA and 17% (v/v) ethanol. The solution was incubated overnight at 4°C and centrifuged at 30,000 ×*g* for 30 min to recover the pellet. This was solubilized and centrifuged at 30,000 ×*g* for 30 min. The supernatant containing the oligogalacturonides was recovered, dialyzed against ultrapure water in a dialysis tube with a molecular mass cut‐off of 1,000 Da (Spectra/Por®) and lyophilized.

Four‐week‐old tomato plants were treated with aqueous oligogalacturonide solution (50 μg/ml in milliQ water) either in leaves or roots. A time course analysis of the response was performed by harvesting leaf material at 1, 6 and 24 h after treatments. For leaf treatments (LT‐), the fourth true leaf of each plant was sprayed with the oligogalacturonide solution using an aerograph until running off. Control treatments were carried out with water (CT‐). Plastic was used to cover the rest of the plant during spraying to avoid contact of the solution with other plant parts. Water was applied similarly for the control treatment. Treated leaves were harvested at the different time points after treatment for the study of local responses (LT‐TL). The sixth fully developed untreated leaf of each plant was also harvested to study systemic leaf responses (LT‐SL), and the untreated roots were harvested to study root systemic responses to leaf treatments (LT‐Root). For the root treatments (RT‐), roots were incubated in a 50 μg/ml oligogalacturonide solution for 1 hr, water was used as control treatment. As for leaf treatments, different plant parts were harvested at 1,6 and 24 h following the incubation. Treated roots were harvested for local responses (RT‐Root). The sixth fully developed untreated leaves were also harvested to study systemic responses in shoots upon OG root treatments (RT‐SL). (Figure S1).

### 2.3 Phytohormones quantification. LC‐ESI tandem mass spectrometry

Six independent plants were harvested and stored at −80°C. The samples were freeze dried and powdered for subsequent analysis. Fifty milligrams of dry powder were used for hormonal extraction. Ultrapure water (Millipore, www.merckmillipore.com) was added containing a pool of internal standards abscisic acid‐d6 (ABA‐d6), salicylic acid‐d5 (SA‐d5) and jasmonate isoleucine‐d6 (JA‐Ile‐d6). Precise quantification was performed by using external calibration curves with each pure chemical compound. The content of the tube was vortexed and left at 4°C in order to hydrate the plant sample. Five glass beads (2 mm Ø) were added into each microtube and the extraction was performed in a mixer mill at a frequency of 30 Hz for 3 min. Tubes were centrifuged at 13,000 rpm for 30′, and supernatant was recovered and placed into a new tube. A second extraction was then conducted, and the supernatant was added to the previous one. The pH was adjusted to 2.5–2.7 with acetic acid and the extraction was partitioned twice against diethyl ether. The two organic fractions were concentrated until dryness in a centrifugal evaporator (Speed vac) at room temperature. Samples were resuspended in 1 ml of H_2_O/MeOH (90:10) with 0.01% of HCOOH leading to a final concentration of internal standards of 100 ng/ml. The chromatographic separation was carried out by injection of 20 μL on an UPLC Kinetex 2.6 μm particle size EVO C18 100 A, 50 × 2.1 mm (Phenomenex). The quantification of the plant hormones was done in an Acquity ultraperformance liquid chromatography system (UPLC; Waters, Mildford, MA), which was connected to a triple quadrupole mass spectrometer (TQD, Waters, Manchester, UK). The chromatographic and mass spectrometry conditions were those published by Gamir, Pastor, Cerezo, and Flors (2012). Masslynx v 4. 1(Waters, Manchester, UK) software was used to process the quantitative data obtained from calibration standards and samples.

### 2.4 LC‐ESI full scan mass spectrometry

The metabolomic analysis was carried out with six biological replicates per treatment. Fifty milligrams of freeze‐dried leaf or root material were extracted at 4°C with 1 ml of MeOH:H_2_O (10:90) containing 0.01% of HCOOH. After the centrifugation at full speed at 4°C for 15 min, the supernatant was filtered through 0.2 μm cellulose filters (Regenerated Cellulose Filter, 0.20 μm, 13 mm D. pk/100; Teknokroma). Twenty microlitres were injected into an Acquity UPLC system (Waters, Mildford, MA) interfaced with a hybrid quadrupole time‐of‐flight instrument (QTOF MS Premier). Subsequently, a second fragmentation function was introduced into the TOF analyser to identify the signals detected. This function was programmed in a t‐wave ranging from 5 to 45 eV to obtain a fragmentation spectrum of each analyte (Gamir, Pastor, Kaever, Cerezo, & Flors, 2014). To elute analytes, a gradient of methanol and water containing 0.01% HCOOH was used. Six independent biological replicates per treatment were randomly injected. The LC separation was performed using an UPLC Kinetex 2.6 μm particle size EVO C18 100 A, 50 × 2.1 mm (Phenomenex). Chromatographic conditions and solvent gradients and further were established as described by Gamir et al. (2014).

### 2.5 Full scan data analysis

Positive and negative electrospray ionization (ESI) signals were analysed independently to obtain a global view of the data conduct. For ESI positive, the instrument detected 5,927 signals and, for ESI negative, 2,962 signals. The data files raw acquired with the Masslynx 4.1 software (Masslynx 4.1, Waters) were transformed into .cdf files with Databridge tool. Chromatographic data files were processed using the software R (http://www.r-project.org/). The XCMS algorithm (www.bioconductor.org; Smith, Want, O'Maille, Abagyan, & Siuzdak, 2006) was used to obtain the peak peaking, grouping and signal corrections. Metabolite amounts were analysed based on the normalized peak area units relative to the dry weight. To test the metabolomic differences between treatments, a nonparametric Kruskal–Wallis test (*p* < .01) was done. Partial least square discriminant analysis and heat map analysis were performed with the metaboAnalyst 4.0 (Chong et al., 2018). Adduct and isotope correction, filtering, clustering, exact mass mapping and metabolic pathway exploration was carried out with the packages MarVis filter, MarVis cluster and MarVis pathway that are integrated in the Marvis suit 2.0 (Kaever et al., 2014). Metabolite identification was carried out based on exact mass accuracy and fragmentation spectra matching with different online database. The database kegg (https://www.genome.jp/kegg/) was used for exact mass identity and for fragmentation spectrum analysis, the Massbank and the Metlin databases were used (www.massbank.jp; www.masspec.scripps.edu).

### 2.6 Quantitative RT‐PCR analysis

The expression of marker genes for the different defence related pathways was analysed by qRT‐PCR using the gene specific primers shown in Table S1. Total RNA from leaves and roots was extracted using Tri‐Sure (Bioline, London, UK) according to the manufacturer's instructions. The RNA was treated with NZY DNase I (NZYtech, Portugal), purified through a silica column using the RNA clean and concentrator™ (Zymo Research, Irvine, CA) and stored at −80°C until use. The first‐strand cDNA was synthesized with 1 μg of purified total RNA using the Primescript™ RT master mix (Takara, Japan) according to the manufacturer's instructions. The qRT‐PCR was conducted using the StepOnePlus™ (Applied Biosystem). Six independent biological replicates were analysed per treatment. We measured the expression of three different housekeeping genes, actin (Solyc03g078400), elongation factor 1‐α (Solyc06g005060) and β‐tubulin (Solyc04g081490) and, to find the optimal normalization gene among these three, we used the Normfinder software (https://moma.dk/normfinder-software). According to the results, expression values were normalized using the housekeeping gene elongation factor 1‐α (EF‐1α), and relative quantification of specific mRNA levels was performed using the comparative 2–Δ(Δ*Ct*) method (Livak and Schmittgen, 2001).

### 2.7 Leucyl‐aminopeptidase (LAP) and β‐1,3‐glucanase activity assays

For protein extraction, 50 mg of fresh plant material were extracted in the extraction buffer (50 mM TRIS–HCl, 0.5 mM MnCl_2_, pH 8). One millilitre of the extraction buffer was added to each sample and was centrifuged for 20 min at 10,000 *g*, 4°C. The supernatant was recovered and stored at −20°C. For LAP activity, Leu‐p‐nitroanilide (Sigma) was prepared from the stock solution as enzyme substrate at a concentration of 3 mM in a solution of 50 mM TRIS‐MnHCl_2_. The stock solution was previously prepared at 150 mM in ethanol and stored at −20°C. To carry out the analysis, 40 μL of the protein sample and 200 μL of the substrate were incubated for 15 min at 37°C and absorbance was measured at 410 nm as described in (Chao, Pautot, Holzer, & Walling, 2000). β‐1,3‐glucanase activity was measured by the Somogy‐Nelson method as described by Román et al. (2011).

### 2.8 *Botrytis cinerea* infection

The fungus was cultivated in potato dextrose agar plates, supplemented with freeze‐dried tomato leaves. Three weeks later, *B. cinerea* spores were collected from plates in 0.5X potato dextrose broth as previously described (Sanmartín et al., 2020).

Four‐week‐old tomato plants were treated with an aqueous solution of OGs in leaves (200 and 50 μg/ml) or roots (50 μg/ml). Six hours after the treatment, treated leaves (for local responses) and the sixth fully developed untreated leaf (for systemic responses) of each plant were detached for the pathogen bioassays. Leaf inoculation was performed applying to the detached leaves 6 μL drops containing a conidia concentration of 5 × 10^6^ spores/ml. In total, we used the sixth fully developed leaf of 10 tomato plants. We inoculated four leaflets per leaf and we applied two drops per leaflet. Leaves were maintained in hermetically sealed boxes with 100% of humidity at 21°C in darkness. Necrotic lesions were evaluated after 5 days.

### 2.9 Statistical analysis

For the hormonal analysis and infection assays data a *t*‐test was conducted using Microsoft office Excel. For qPCR data and enzymatic activity, a one‐way ANOVA was used to find overall differences among the expression levels. Post hoc LSD was used to find significant differences among treated and control plants (*p* < .05). All the metabolome profiling data were analysed using a Kruskal–Wallis analysis provided in MarVis suite 2.0.

## 3 RESULTS

### 3.1 Treatment with OGs triggers local and systemic hormone responses in tomato roots and leaves

The spatio‐temporal regulation of local and systemic responses to OGs was analysed on tomato plants grown in a hydroponic system. An aqueous OG solution was applied to the fourth fully expanded leaf (leaf treatment; LT) or to the roots (root treatment; RT). Local responses were examined in the treated organs (local, leaves or roots). In the case of leaf treatment, systemic responses were analysed in roots and in the non‐consecutive, fully developed younger leaf, attending to the vascular connection in tomato plants (Orians, Pomerleau, & Ricco,^2000^) (sixth true leaf). In the case of root treatment, the equivalent sixth true leaf was harvested to study systemic responses (Figure S1).

**FIGURE 1:**
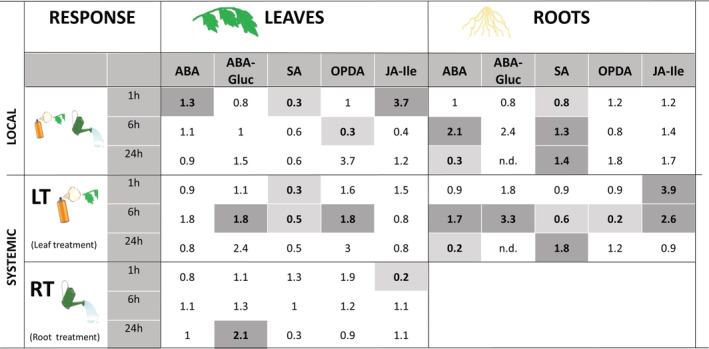
Time course analysis of local and systemic changes in hormonal levels upon OG treatment in leaves or roots of tomato plants. Hormonal levels were quantified by UPLC‐MS/MS at 1, 6 and 24 hr in tomato plants elicited with OGs solution of 50 μg/ml. For leaf treatments (LT), the fourth true leaf was sprayed with the OGs solution and samples from the treated leaf (local response‐leaves‐), upper leaf and roots (systemic response) were harvested at the different time points. For root treatments (RT) the OG solution was applied to roots and samples at the different time points were taken from roots (local response‐roots‐) and in the upper leaves for systemic responses (Systemic, RT). Hormone contents are shown in Figure S2a–c. Here, numbers represent fold induction in the hormone levels of treated vs control plants. Bold numbers indicate significant differences (*t*‐test; *p*‐value < .05; *n* = 6). Dark shading cells highlight significantly over accumulated compounds and, light shading cells highlight compounds that are significantly less accumulated

First, we studied the changes in the main plant defence hormones and some precursors and derivatives (SA, ABA, ABA glucoside [ABA‐Gluc], JA‐Ile and 12‐oxo‐phytodienoic acid [OPDA; JA precursor]). The levels of these compounds were measured at 1, 6 and 24 hr post treatment (hpt) and the data are presented in Figure S2a–c. A summary of the changes, expressed as fold changes compared to the corresponding mock‐treated controls is shown in Figure 1.

Local responses to OGs appear to be very different in leaves and roots (Figure 1). In treated leaves, changes were observed mainly at 1 hpt, consisting of a slight accumulation of ABA, an important accumulation of JA‐Ile (3.7 fold compared to mock‐treated leaves) and a decrease of SA. A decrease of OPDA at 6 h was the only significant later response. In OG‐treated roots only SA showed a change at 1 hpt: a slight decrease, followed by a moderate increase at later time points. ABA transiently increased at 6 hpt, dropping below control levels at 24 h.

Systemic responses of leaves to OGs were also different depending on the type of treatment (root or leaf) (Figure 1). A decrease of SA at all time‐points and an accumulation of OPDA and ABA‐Gluc levels at 6 hpt were observed only upon leaf treatment, whereas a transient decrease of JA‐Ile was observed exclusively in RT‐SL at 1 hpt. Levels of ABA‐Gluc increased in systemic leaves with both treatments, with a delay in the case of root treatment.

Notably, systemic changes in hormone levels in the roots of leaf‐treated plants were more pronounced (Figure 1). A strong accumulation of JA‐Ile occurred at 1 hpt that was maintained up to 6 hpt and paralleled with a reduction of the JA precursor OPDA, revealing an upregulation of the oxylipin pathway in the roots upon leaf treatment. ABA and its derivative ABA‐Gluc also showed a systemic transient increase in roots, and SA levels increased at the latest time point.

These data show that tomato plants respond systemically to OG treatment in leaves, with the most conspicuous hormone changes occurring in the root. Regardless of the site of OG treatment (leaf or root), an early and transient increase of ABA and a late increase in SA occur mainly in roots. Instead, induction of the oxylipin pathway, shown as early increases in JA‐Ile levels, occurs in both leaves and roots only upon leaf treatments, although the effect is more durable in roots.

This hormone analysis was complemented with the expression analysis of genes involved in the JA, SA, ABA and ET biosynthesis pathways (Table 1). In the local response, we observed an early and transient upregulation of the JA biosynthetic gene *LOXD* in both leaves and roots. In the systemic response, *LOXD* was induced only upon leaf treatment, with a stronger and more sustained induction in the roots than in upper leaves, confirming a general activation of the oxylipin pathway upon leaf treatment. In contrast, *PAL* (SA biosynthesis gene and the first step in the phenylpropanoid biosynthetic pathway) was only locally up‐regulated in roots. As a systemic response, *PAL* was up‐regulated in roots after leaf treatment and transiently in leaves after root treatment. Finally, induction of the ABA biosynthetic gene *NCED* was found mostly in roots as both a local and a systemic response to leaf treatment.

**TABLE 1:** Transcriptional regulation of phytohormone biosynthetic genes upon OG treatment

		Leaves				Roots			
Response	Time (hr)	*LOXD*	*PAL*	*NCED*	*ACO1*	*LOXD*	*PAL*	*NCED*	*ACO1*
Local	1	***6.4***	1.2	1.3	***10***	***9***	***5.3***	***6.5***	***12.8***
	6	0.8	**0.5**	0.7	1	0.8	***2.8***	1.7	***5.3***
Systemic (LT)	1	***6***	0.8	0.8	***2.1***	***62***	***2.6***	***25.5***	1.3
	6	1.3	0.7	1.2	1.3	***3.3***	***2.3***	***2.1***	***3.6***
Systemic (RT)	1	1	***2.2***	1	0.6	–	–	–	–
	6	**0.5**	**0.6**	0.8	0.7	–	–	–	–

*Notes:* Quantitative RT‐qPCR analysis of *LOXD* (lypoxigenase D, involved in JA biosynthesis), *PAL* (phenylalanine ammonia lyase, involved in SA biosynthesis), *NCED* (9‐cis‐epoxycarotenoid dioxygenase, involved in ABA biosynthesis) and *ACO1* (ACC Oxidase 1, coding for the enzyme responsible for the limiting step in ET biosynthesis) in leaves and roots of OG treated plants. Changes related to local responses were evaluated in treated leaves and roots. Systemic responses were evaluated in roots and upper leaves upon OG treatment in leaves (LT) or in upper leaves upon OG treatment in roots (RT). Numbers correspond to the fold induction of the gene expression levels in treated vs control plants (*n* = 6 from six biological replicates). Values are normalized relative to the tomato housekeeping gene EF‐1α. Bold numbers indicate significant differences and cells in italics highlight inducible values (one‐way ANOVA; LSD; *p*‐value < .05; *n* = 6).

The role of ET in DAMPs signalling has been previously described (Díaz et al., 2002; O'Donnell et al., 1996; Simpson et al., 1998). For example, OGs treatments in tomato and Arabidopsis seedlings boost local ET levels and ET biosynthetic genes (Gravino et al., 2015; Simpson et al., 1998). Here, we examined the expression of the gene *ACO1*, well defined marker of ET pathway encoding the ACC oxidase 1, responsible of the limiting step in ET biosynthesis (Jafari, Haddad, Hosseini, & Garoosi, 2013). *ACO1* was markedly up‐regulated as a local response in both leaves and roots. The induction was transient in OG‐treated leaves, but stronger and more sustained in OG‐treated roots. As a systemic response, *ACO1* was induced in leaves only upon leaf treatment, showing a similar regulation pattern than the JA biosynthetic gene. Systemic induction in the roots was also observed upon leaf treatment. Thus, gene expression analyses confirm the activation by OG treatment of JA, ABA and ET signalling, with varying patterns according to the application site. They also support the conclusion of a strong response to OGs in roots, either as a local or a systemic response (Table 1).

As a whole, the transcriptional and metabolic data reveal that OG treatment impacts hormone signalling both at a local and a systemic level with induction of the oxylipins, ABA and ET pathways. Local hormone‐related responses to OGs in leaves occur early and transiently, while systemic responses are delayed compared to local ones, with the stronger effects detected at 6 hpt. Remarkably, changes in hormone levels and expression of hormone biosynthetic genes were, in general, stronger and more sustained during time in roots.

### 3.2 OG perception modulates tomato plant metabolism in an organ‐specific manner

To characterize the responses elicited by OGs in tomato, we decided to carry out a non‐targeted metabolomic analysis at 6 hpt, the time point showing the major changes according to the hormone profiles (Figure 1). For a global perspective, we conducted an unsupervised principal component analysis (PCA) (Figure S3). The component 1, which explains the highest source of variability, clearly differentiated between root and leaf samples, coherent with specific metabolomic profiles in the different organs. Within leaves, variability was mainly related to the leaf age, since older leaves (fourth leaf, chosen for OG local treatment) and younger/upper leaves (true sixth leaf, chosen for the analysis of the systemic response) displayed very distinctive metabolic profiles, despite of being both adult, fully expanded leaves. Regardless of this difference, responses to OGs in leaves appeared much subtler than responses in roots (Figure S3). In roots, local and systemic responses to OGs showed marked differences (Figure S3).

**FIGURE 2:**
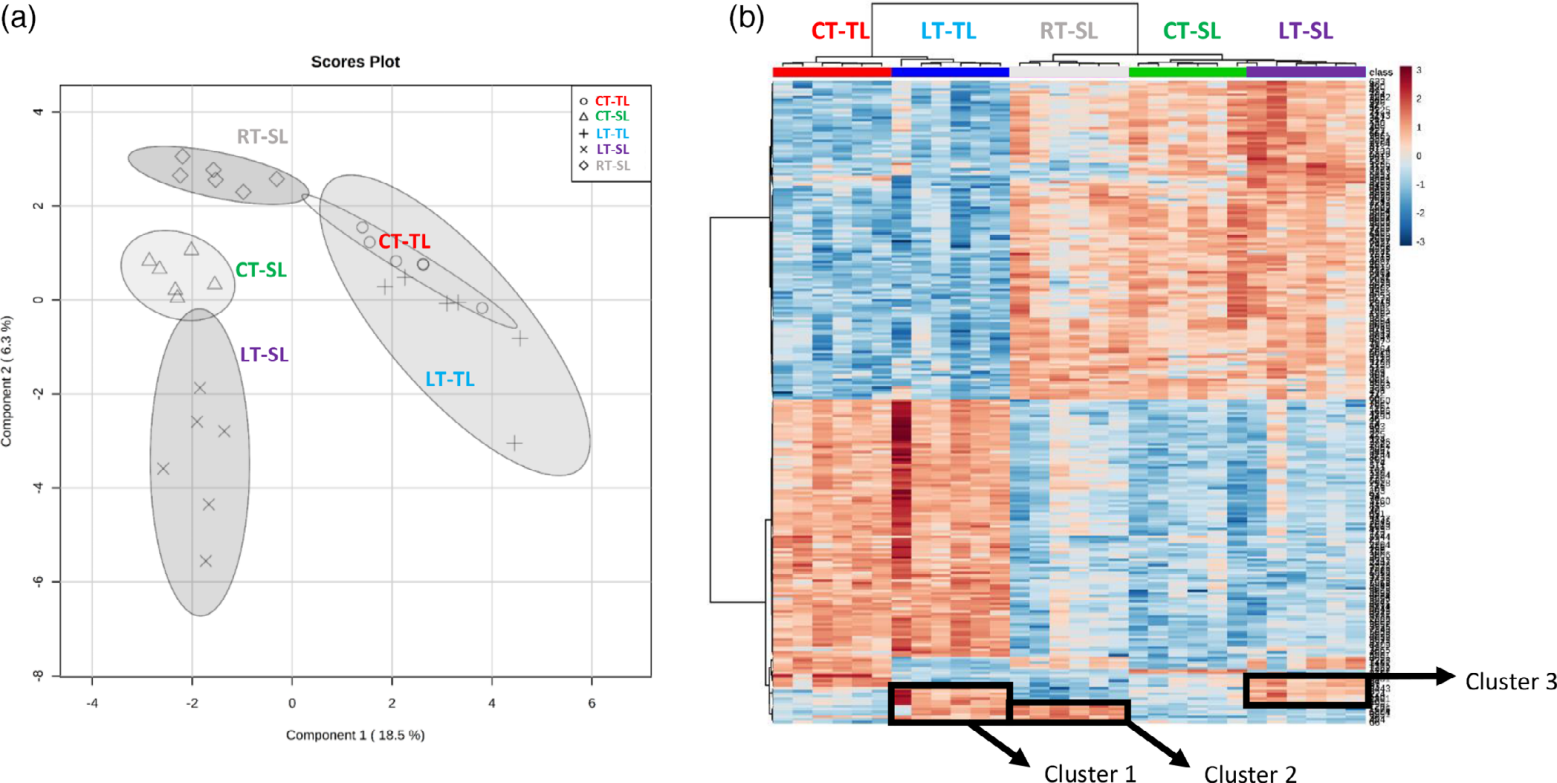
Impact of OG treatment on the leaf metabolic profiles (a) sPLS‐DA representation of ESI− and ESI+ signals obtained from a non‐targeted analysis by UPLC‐QTOF to monitor metabolomic changes 6 hr after OGs treatments. Three‐week‐old plants were treated in leaves or roots with a 50 μg/ml solution of OGs. Leaf samples were harvested 6 hr post treatment. Data points represent six biological replicates injected randomly into the UPLC‐QTOF. The signals corresponding to different treatments were compared using the non‐parametric Kruskal–Wallis test, and only data with a *p*‐value < .01 between groups was used for subsequent processing. (b) Heatmap analysis of leaf metabolites responding to OGs. Signals from ESI+ and ESI− with *p*‐value < .01 were used to generate the heatmap analysis. The top 250 signals with the lowest *p*‐value were selected to represent the heatmap. The relative amount of the metabolites was determined in all the samples by normalizing the chromatographic pick area of each compound with the dry weight of the corresponding sample. For control‐treated plants with water (CT‐) we analysed treated leaf response (CT‐TL) and systemic leaf response (CT‐SL). For OG leaf‐treated plants (LT‐) we analysed treated leaf responses (LT‐TL) and systemic leaf responses (LT‐SL). Finally, for OG root‐treated plants (RT‐) we analysed systemic leaf responses (RT‐SL)

For an exploratory approach, we decided to perform a sparse partial least square discriminant analysis (sPLS‐DA). Variable selection in the sPLS‐DA improves classification accuracy and characterization compared to classical PCA (Lê Cao, Boitard, & Besse, 2011). A sPLS‐DA in leaves showed that OG treatments had a stronger systemic than local impact in the metabolic profile (Figure 2a). The systemic response was also different when OG treatment was applied to leaves or roots. A heatmap analysis was carried out to identify the main features responsible for such differential profiles (Figure 2b). Different clusters showing signals with higher intensity due to OG treatments were selected for signal identification. Cluster 1 was selected to study local responses in leaves (LT‐TL) and cluster 2 and 3 were selected for systemic responses: Cluster 2 for systemic responses in leaves after root treatments (RT‐SL) and cluster 3 for systemic responses in leaves after leaf treatment (LT‐SL). Tentative identification, based on exact mass accuracy and on‐line fragmentation spectra of the signals was performed, and the pathways of the identified metabolites that are differentially accumulated in the different treatments are shown in Table 2. Remarkably, while almost 6 and 3.5% of the total detected signals were significantly more accumulated in roots as a local or systemic response to OGs, respectively, only about 2% were significantly more accumulated in the local or systemic responses in leaves.

**TABLE 2:** Pathways of identified metabolites differentially accumulated locally or systemically upon OG treatment in leaves or roots

Response	Leaves	Roots
Local	(**2%**)	(**5.8%**)
	Flavonoid biosynthesis (**3**)	Flavonoid biosynthesis (**4**)
	Porphyrin and chlorophyll metabolism (**2**)	Lignan (**3**)
		Alkaloids biosynthesis (**3**)
		Auxin degradation (**2**)
		Biotin metabolism (**2**)
		Terpenoids‐quinone biosynthesis (**2**)
		Lysine biosynthesis (**2**)
Systemic (LT)	**(1.8%)**	**(3.4%)**
	Flavonoid biosynthesis (**3**)	Amino acid metabolism (**7**)
	Purine metabolism (**2**)	Dipeptide (**3**)
	Amino acid metabolism (**2**)	Lignan (**2**)
	Fatty acid biosynthesis (**2**)	
Systemic (RT)	**(2.2%)**	
	Alkaloids biosynthesis (**3**)	
	Amino acid metabolism (**3**)	

*Notes:* The percentage of signals showing significantly higher accumulation from the total identified signals for a given treatment is shown in brackets. Identified metabolites were assigned to their corresponding metabolic pathways, and the number in the right refers to the identified compounds within that pathway. The signals corresponding to different treatments were compared using the non‐parametric Kruskal–Wallis test, and only data with a *p*‐value < .01 between groups were used for subsequent identification.

Regarding the metabolic pathways, local leaf responses to OGs included changes in flavonoid biosynthesis and in porphyrin‐chlorophyll metabolism (Table 2). Considering systemic leaf responses to root and leaf treatment, the comparison between cluster 2 (systemic, RT) and cluster 3 (systemic, LT) revealed that none of the signals tentatively identified were shared, explaining the difference observed in the sPLS‐DA. Systemic leaf responses upon leaf treatment included flavonoid accumulation and changes in purine, amino acid and fatty acid biosynthesis. In contrast, systemic leaf responses to root treatment mostly involved tropane alkaloids, although it also impacted amino acids metabolism (Table 2). Remarkably, two of the three putative identified amino acids belong to the arginine and proline metabolism, known precursors of the tropane alkaloids biosynthesis.

**FIGURE 3:**
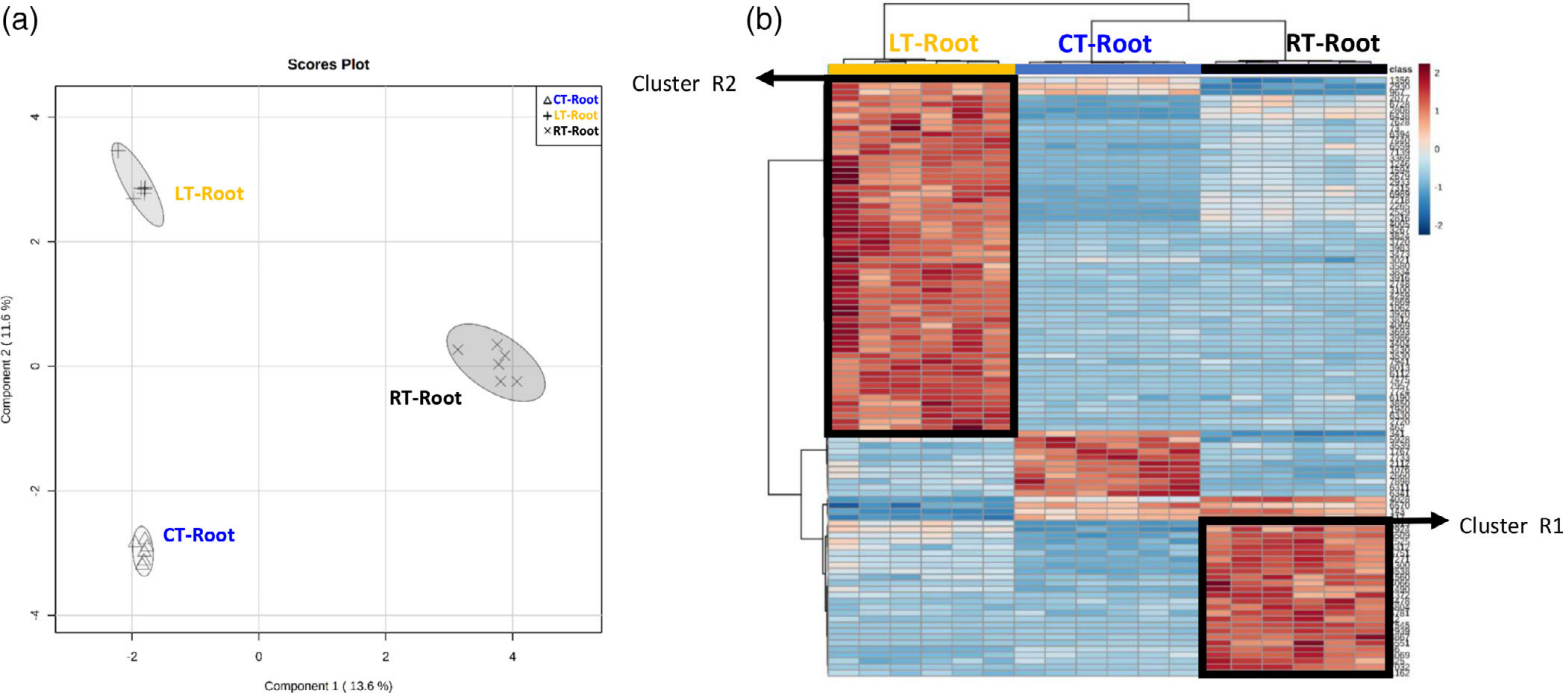
Impact of OG treatment on the root metabolic profiles (a) sPLS‐DA representation of ESI− and ESI+ signals obtained from a non‐targeted analysis by UPLC‐QTOF‐MS to monitor metabolomic changes 6 hr after OGs treatments. Three‐week‐old plants were treated in leaves or roots with a solution of OGs 50 μg/ml. Root samples were harvested 6 hr post treatment. Data points represent six biological replicates injected randomly into the UPLC‐QTOFMS. The signals corresponding to different treatments were compared using the non‐parametric Kruskal–Wallis test, and only data with a *p*‐value < .01 between groups was used for subsequent processing. (b) Heatmap analysis of root metabolites responding to OGs. Signals from ESI+ and ESI– with *p*‐value < .01 were used to generate the heatmap analysis. The top 100 signals with the lowest *p*‐value were selected to represent the heatmap. The relative amount of the metabolites was determined in all the samples by normalizing the chromatographic pick area of each compound with the dry weight of the corresponding sample. For control‐treated plants with water (CT‐) we analysed root responses (CT‐Root). For OG root‐treated plants we analysed local root responses (RT‐Root) and for OG leaf‐treated plants we analysed systemic root responses (LT‐Root)

In roots, the impact of OGs on the tomato metabolome is much stronger compared to that in leaves (Figure 3a). The heat‐map analysis (Figure 3b) allowed us to pinpoint the cluster R1, corresponding to metabolites that strongly accumulated as a root local response to OG (RT‐Root), and the cluster R2, corresponding to metabolites that accumulated in roots as a systemic response to leaf treatment (LT‐Root). Tentative identification of the features in cluster R1 revealed that roots respond to OGs by accumulating flavonoids, alkaloids and lignans, all well‐known antimicrobial metabolites (Table 2). In addition, auxin, biotin, lysine and terpenoids‐quinone biosynthesis were also accumulated locally in treated roots. Noteworthy, biotin and lysine metabolism are related to the alkaloid biosynthetic pathways (Kegg pathway map00780). As a systemic root response to leaf treatment (cluster R2) lignans also accumulated, but mostly the amino acid metabolism was affected.

In summary, flavonoids accumulation was a common response to OGs in roots and shoots. Alkaloids accumulated only following root treatments, either locally in roots (RT‐Root) or systemically in leaves (RT‐SL), indicating that roots lead the responses related to alkaloids accumulation in all plant organs. As an example, the alkaloid putatively identified as anatalline accumulated in both roots and leaves of root treated plants (Figure S4), but did not change upon leaf treatment. On the other hand, lignans exclusively increase in roots both as local and systemic response, pointing to lignan accumulation as a common root response to OGs, regardless the site of perception. However, signals showing higher changes in the systemic root response were from the primary metabolism, suggesting a reorganization of root metabolism after the perception of danger signals in leaves.

### 3.3 Flavonoids and alkaloids biosynthetic genes are up‐regulated in tomato roots as a local and systemic response to OGs

Flavonoids and alkaloids, both accumulated in response to OGs, are phenylpropanoids derivatives that significantly contribute to plant resistance (Mithöfer & Boland, 2012; Treutter, 2005). To gain further insight into the regulation of flavonoids and alkaloids in response to OGs, we studied changes in the expression of relevant biosynthetic genes at the local and systemic level 1 and 6 hr after leaf or root treatment (Table 3). For the analysis, we chose *CHALCONE SYNTHASE 1* (*CHS*.*1.1*), encoding the first enzyme of the flavonoid biosynthetic pathway that produces naringenin chalcone, *CHALCONE ISOMERASE 1* (*CHI1.1*), responsible for the downstream reaction that converts naringenin chalcone to naringenin, and *PUTRESCINE N‐METHYLTRANSFERASE* (*PMT*), encoding a key enzyme in the biosynthesis of tropane alkaloids (Biastoff, Brandt, & Dräger, 2009; Petrussa et al., 2013).

**TABLE 3:** Transcriptional regulation of flavonoids and alkaloids biosynthetic genes upon OG treatment

		Leaves			Roots			
Response	Time (hr)	*CHS1.1*	*CHI1.1*	*PMT*	*CHS1.1*	*CHI1.1*	*PMT*	
Local	1	0.9	1	nd	**0.1**	**0.5**	**0.1**	
	6	**0.4**	**0.4**	nd	***59***	***5***	***7***	
Systemic (LT)	1	**0.4**	0.7	nd	**0.1**	1	1.1	
	6	0.7	0.9	nd	0.4	***2.6***	***3.6***	
Systemic (RT)	1	0.8	1	nd	–	–	–
	6	**0.5**	**0.6**	nd	–	–	–

*Notes:* Quantitative RT‐qPCR analysis of *CHS1.1*, *CHI1.1* (flavonoid biosynthesis genes) and *PMT* (alkaloid biosynthesis gene) confirms upregulation of such metabolic pathways in roots in response to OGs treatments. Numbers correspond to the fold induction of the gene expression levels in treated vs control plants (*n* = 6 from six biological replicates). Values are normalized relative to the tomato housekeeping gene EF‐1α. Expression levels of PMT in leaves were not detectable (nd). Bold numbers indicate significant differences and cells in italics highlight inducible values (one‐way ANOVA; LSD; *p*‐value < .05; *n* = 6).

In leaves, none of those genes showed any upregulation in the gene expression level in response to OGs at any of the timepoints tested regardless the OG application site. No expression of *PMT* was detected in leaves. In contrast, the expression of these genes showed significant changes in roots both as a local and a systemic response to the treatment. *CHI1.1* and *PMT* were induced in roots as a systemic response to OG treatment in leaves (LT‐Root). Interestingly, all three genes were down‐regulated locally in roots 1 hpt and strongly up‐regulated upon root treatment 6 hpt, with a particularly strong effect for *CHS1.1* (59 fold). These results are in agreement with the metabolic analyses and collectively evidence the key role of roots in flavonoid and alkaloid biosynthesis following OG perception (Table 3).

### 3.4 OGs induce systemic resistance to *Botrytis cinerea*

The strong impact of OG treatment in systemic tissues at very early time‐points prompted us to investigate whether OGs induce systemic resistance in tomato plants against the necrotrophic pathogen *B. cinerea*.

**FIGURE 4:**
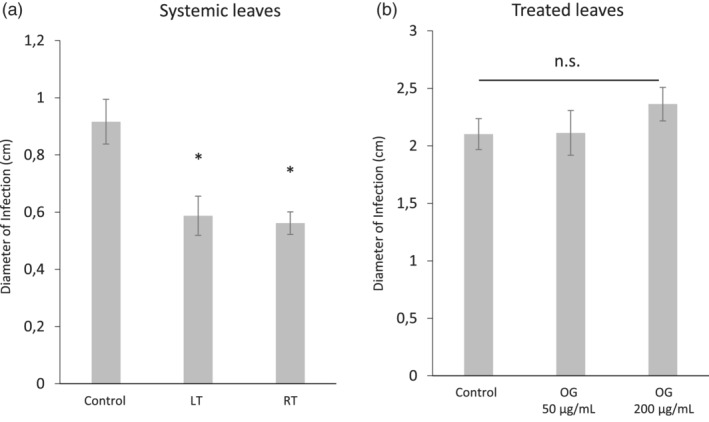
OG treatments induce systemic resistance against *Botrytis cinerea*. Three‐week‐old tomato plants were treated in roots with a 50 μg/ml OGs solution or in leaves with 50 μg/ml or 200 μg/ml OGs solution. Six hours after the treatment, plants were drop inoculated in upper or treated leaves with a *Botrytis cinerea* conidia suspension of 5 × 10^6^ spore × ml^−1^. Lesion diameter was measured 5 days after inoculation. Data presented shows the average lesion diameter ± SE (*n* = 10 plants). Systemic leaf responses were analysed comparing upper untreated leaves from water‐treated plants as control to upper leaves from OGs‐ leaf treated plants (LT) and to upper leaves from OGs‐root treated plants in roots (RT). Asterisks indicate statistically significant differences compared to control plants (* water treated; *t*‐test; *p* < .05)

Systemic protection was examined by treating tomato roots or leaves with 50 μg/ml of OGs and infecting at 6 hpt (the untreated true sixth leaf) with *B. cinerea*. Both root and leaf treatments (LT and RT) led to a significant reduction of the necrotic lesions compared to the corresponding control treated leaves (CT‐SL), confirming that systemic responses to OG treatment are associated to systemic resistance against this pathogen in tomato. Remarkably, the protection level was similar regardless of the site of OGs application (roots or leaves) (Figure 4a).

Local OG‐induced resistance was also examined, by inoculating the OG‐treated leaves (LT‐TL) at 6 hpt. No reduction of the necrotic symptoms was observed compared to control‐treated leaves (CT‐TL) at either 50 μg/ml or 200 μg/ml OGs (Figure 4b). Thus, under our experimental conditions and at the time point examined (6 hpt) OG treatment in tomato induced systemic but not local resistance to *B. cinerea*.

### 3.5 Antifungal defences are up‐regulated as a systemic response to OGs

**FIGURE 5:**
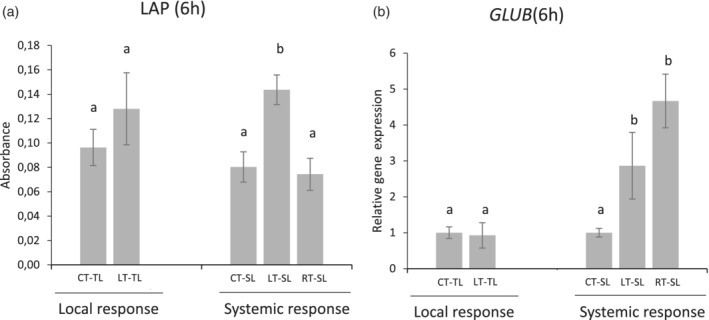
OG perception in leaves increases systemic leucyl aminopeptidase activity and β‐1,3‐glucanase *GluB* gene expression. Leucyl aminopeptidase activity (JA‐responsive protein) and quantitative RT‐qPCR analysis of *GluB* (coding for a pathogen inducible β‐1,3‐glucanase). Local responses were analysed in water‐treated (CT‐TL) or OG‐treated leaves (LT‐TL). Systemic responses were analysed comparing upper untreated leaves from water‐treated plants as control (CT‐SL) to upper leaves from OG‐leaf treated plants (LT‐SL) and to upper leaves from OG‐root treated plants (RT‐SL). Bars represent mean ± SD, *n* = 6 from six biological replicates. Different letters indicate statistically significant differences compared to control plants (one‐way ANOVA; LSD; *p*‐value < .05)

Plant resistance to pathogens is generally the result of a combination of different defence mechanisms. Aiming to understand why the OG treatments induced systemic—but not local—pathogen resistance, we explored other potential antifungal defence responses that may contribute to the enhanced resistance against *B. cinerea*. Hence, we analysed the well characterized pathogenesis related proteins Leucyl aminopeptidase (LAP) and β‐1,3‐glucanases. LAP is a JA regulated, wound‐responsive protein, displaying a dual role as aminopeptidase but also as a chaperone (Fowler et al., 2009; Scranton, Yee, Park, & Walling, 2012). β‐1,3‐glucanases, are inducible enzymes with antimicrobial properties for their action on the β‐glucans in fungal cell walls. LAP activity was induced only in distal leaves in response to leaf OG treatment (LT‐SL), whereas no changes were observed locally in treated nor upon root treatment (Figure 5a). Total β‐1,3‐glucanase activity did not vary among the different treatments (Figure S5). Several β‐1,3‐glucanases are present in tomato with differential regulation patterns, and total enzymatic activities may mask enhancement of specific glucanase isoforms. Thus, we examined the expression levels of *GluB*, encoding a β‐1,3‐glucanase, known to be inducible by JA, ET and pathogens (van Kan, Joosten, Wagemakers, van den Berg‐Velthuis, & de Wit, 1992). While *GluB* expression was not induced in OG‐treated leaves (local response), it was boosted in leaves as a systemic response to OG treatments in both leaves or roots treatments (Figure 5b). Note that the enhanced *GluB* expression correlates with the tissues showing OG induced resistance in the pathogen bioassay.

**FIGURE 6:**
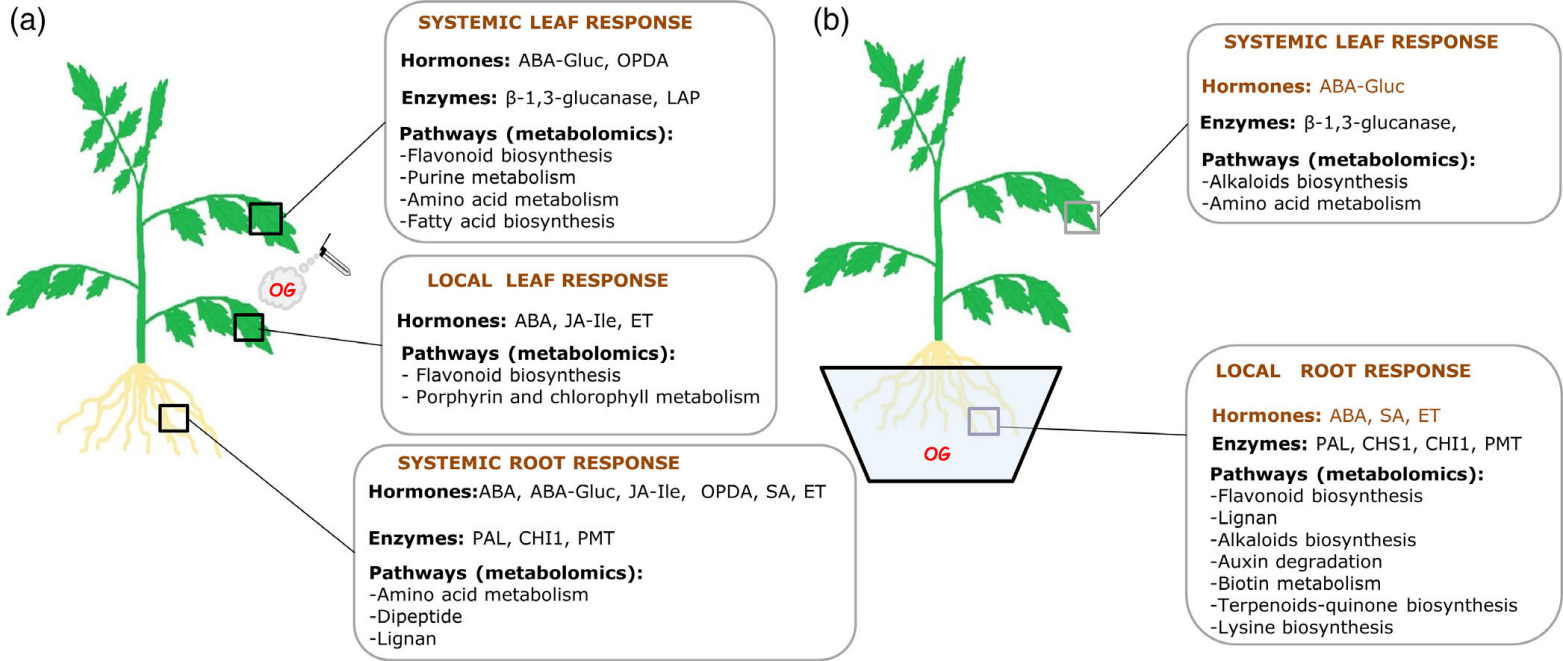
Summary of the responses activated in tomato leaves and roots upon OG treatments. The model displays local and systemic tomato responses at the hormonal, enzymatic and metabolomic level depending on the organ of the perception. (a) Plant responses to OG treatment in leaves. Local responses in treated leaves and systemic responses in leaves and roots are shown. (b) Plant responses to OG application in roots. Local responses in roots and systemic response in leaves are shown

Taken together, our results suggest that the accumulation of defensive metabolites and the induction of pathogenesis‐related proteins may underlie the efficient systemic protection against *B. cinerea* induced by OGs in tomato (Figure 6).

## 4 DISCUSSION

DAMPs recognition and signalling is one of the earliest events of the plant and animal immune system (Heil & Land, 2014). The relevance of DAMPs signalling in the immune responses has received increasing attention in the last years (De Lorenzo et al., 2018; Gust, Pruitt, & Nürnberger, 2017) but our mechanistic and functional understanding of the process in plants is still very limited. Oligogalacturonides are DAMPs derived from the plant cell wall, and the responses they trigger have been mainly studied in Arabidopsis, at the local level (Davidsson et al., 2017; Gravino et al., 2017). In this study we investigated how tomato plants respond, both locally and systemically, to the perception of OGs in roots and shoots, and whether the systemic response to OGs confers resistance against *B. cinerea*.

Plant responses to OGs have been previously shown to be mediated by hormone signalling. For example, JA mediates some responses to OGs in tomato (Doares et al., 1995) and in Arabidopsis (Davidsson et al., 2017; Denoux et al., 2008; Ferrari, Plotnikova, De Lorenzo, & Ausubel, 2003). Our time‐course analysis of hormone changes in tomato plants after OG recognition reveals a complex regulation pattern both at the local and systemic level during the 24 hr following OG application in roots or shoots. Hormone quantification complemented with gene expression analysis of related biosynthetic genes show the involvement of the JA, ABA and ET signalling pathways in the response to OGs. The activation of these pathways in response to these damage signals is in agreement with their reported regulation during the tomato wound responses (Tian, Peiffer, De Moraes, & Felton, 2014). JA signalling was activated in leaves and roots upon leaf treatment, and ET biosynthesis was activated as a local response to OGs in both roots and leaves. The ABA pathway was also altered, but changes occurred mostly in roots. The speed and magnitude of the responses depended on the organ that perceived OGs. Fast and transient hormone changes occurred locally in the OG‐treated leaves, with a maximum at 1 hpt, while systemic hormone changes in both leaves and roots of leaf‐treated plants were more pronounced at 6 hpt. Notably, systemic hormone changes appeared stronger than local ones, suggesting that, after a fast and transient local response, the plant allocates its resources to defend the undamaged distal tissues.

Remarkably, the untargeted metabolomic analysis showed that the most prominent response occurs in the roots of leaf‐treated plants. Roots are the key regulators of plant defence responses to aboveground challenges; for example, foliar herbivory induces fast changes in roots leading to the synthesis of antiherbivore compounds such as alkaloids (Erb, Lenk, Degenhardt, & Turlings, 2009; Erb, Meldau, & Howe, 2012; Agut, Gamir, Jaques, & Flors, 2016). Metabolic responses to OGs are stronger in roots than in leaves and, in the same line, the proportion of identified compounds over‐accumulating after OG treatment is also higher in roots than in leaves. Similarly, JA accumulation in tomato leaves after the attack of the root‐knot nematode *Meloidogyne incognita* depends on the production of electric signals and ROS accumulation in roots (Wang et al., 2019). In conclusion, our results support that damaged‐self recognition impacts the root metabolic composition altering aboveground responses.

A detailed analysis of the changes in the metabolomics profile showed accumulation of phenylpropanoid compounds such as lignans and flavonoids in response to OGs. Lignans accumulated only in roots both as a local or systemic response and have been recently related to plant defence. Overexpression of a lignan biosynthesis gene in soybean roots induced resistance against the oomycete *Phytophthora sojae* (Li et al., 2017), and we have recently shown that the root derived lignan yatein is involved in mycorrhiza induced resistance against *B. cinerea* in tomato (Sanmartín et al., 2020). In contrast to lignans, flavonoids accumulated in roots only as a local response to OGs, and in leaves as both local and systemic response to leaf treatment. Interestingly, this is not the first indication of flavonoid accumulation after DAMPs perception. For example, foliar application of chitosan oligomers and OGs (COS‐OGA) induced *PAL* in rice roots and shoots, (Singh et al., 2019). Additionally, untargeted metabolomic analyses showed that flavonoids are accumulated in Arabidopsis leaves after NAD+ treatments (Pétriacq et al., 2016). We further found that the flavonoid biosynthetic genes are strongly up‐regulated in the roots, after local or distal OG treatments, but not in leaves. These results are in agreement with the finding that flavonoids synthesized in Arabidopsis roots after OG perception can be transported through the plant (Hernandez‐Mata et al., 2010; Petrussa et al., 2013). Together, we suggest that lignans and flavonoids are synthetized in roots after OG recognition, and flavonoids are transported through the vascular system to the distal parts of the plants.

We also found an accumulation of tropane alkaloids as a local response in roots and as a systemic response in leaves, but only upon root treatment. Indeed, the putatively identified anatalline, a JA‐inducible tropane, piperidine and pyridine alkaloid (Häkkinen et al., 2004) showed elevated levels in roots and leaves of root treated plants, but no change upon leaf treatment. The role of alkaloids in plant defence has been extensively studied against herbivore insects (Agut et al., 2016; Erb et al., 2009), but less evidences are provided on their role against phytopathogens. However, a recent study showed that higher levels of α‐tomatine enhance tomato resistance against *Phytophthora infestans* and *B. cinerea* (Chen, Meng, He, Zhang, & Luan, 2019). Furthermore, we showed that roots displayed a strong increase in *PMT* gene expression—coding for a key enzyme of the tropane alkaloid biosynthesis pathway—as both a local and systemic response to OGs. Interestingly, *PMT* gene expression in leaves was non‐detectable, supporting the notion that roots are responsible for the synthesis of tropane alkaloids, which can be later transported systemically. The synthesis of tropane alkaloids in roots has been reported (Kohnen‐Johannsen & Kayser, 2019), and reciprocal grafting experiments show that the alkaloid patterns in leaves of solanaceae species are determined by the rootstock rather than the foliage (Bais, Sudha, Suresh, & Ravishankar, 2001).

Here, we show that the responses observed upon OG treatments are biologically relevant for defence in tomato, since they confer systemic resistance against the necrotrophic pathogen *B. cinerea*. So far, only one report in Arabidopsis describes systemic protection against this fungus, with no further mechanistic study (Ferrari et al., 2007). Most of the knowledge relates instead to the protection induced by local elicitation with OGs (Aziz et al., 2004; Ferrari et al., 2007; Galletti et al., 2008; Galletti et al., 2011). Thus, our study clearly reveals striking differences between local and systemic defence/resistance responses in tomato. Besides the potentially fungicide compounds systemically accumulated in OG treated plants, such as the alkaloids, we also looked for other potential players that may contribute to the observed OG‐systemic induced resistance against *B. cinerea*. Therefore, we explored the activity or gene expression levels of leucyl aminopeptidase (LAP) and the antimicrobial PR proteins β‐1,3‐glucanase (GluB) (van Kan et al., 1992). *GluB* expression was higher in the tissues showing increased resistance to *B. cinerea* (RT‐SL and LT‐SL), supporting its possible role in OGs‐induced systemic resistance. It is worth noting that high doses of OGs (500 μg/ml) have been shown to induce glucanase activity in grapevine cells (Aziz et al., 2004) and that *GluB* gene expression is up‐regulated in the *Solanum lycopersicoides* Botrytis interaction (Smith, Mengesha, Tang, Mengiste, & Bluhm, 2014). LAP is a JA‐inducible enzyme that plays a key role in tomato plant responses towards biotic attack (Fowler et al., 2009). LAP activity increased upon leaf treatment only in systemic leaves and not in the treated ones. Thus, the induction of both LAP activity and *GluB* expression in systemic leaves, but not in the OG‐treated leaves correlates with the systemic induced resistance observed.

Overall, our work reveals the complexity of the plant responses to damage perception, showing that, upon OG treatment, defence responses are triggered throughout the plant, but they differ depending on the site of DAMP application (summarized in Figure 6a,b). Our results are in agreement with the hypothesis of Tytgat et al. (2013) pointing that roots and aerial organs can activate different signalling cascades, thus likely contributing information about the site of induction. This would provide plants with a mechanism to fine‐tune defence responses according to the damaged organ. The observation that systemic responses are stronger than local ones in both roots and shoots suggests that plants invest more resources in preparing distal tissues for efficient defence activation against a potential upcoming attack (Gómez, Ferrieri, Schueller, & Orians, 2010; Kundu, Mishra, & Vadassery, 2018; Steinbrenner, Gómez, Osorio, Fernie, & Orians, 2011).

In summary, we show that in tomato, responses to OGs trigger enhanced systemic resistance to pathogens. The response involves the regulation of JA, ABA and ET signalling pathways, and the activation of main metabolic pathways for the biosynthesis of antimicrobial metabolites such as alkaloids, flavonoids and lignans. Most of them are likely synthesized in the roots, even when OGs are applied in leaves, but can be later transported from the roots to the shoots. Thus, our wide analyses highlight the key role of roots in coordinating systemic defence responses to damage in plants. Identifying potential mobile signals orchestrating this root/shoot bidirectional dialogue is an exciting challenge for future research.

Our research highlights the need of addressing the spatio‐temporal regulation of plant responses to DAMPs to understand how plants integrate danger signals and shape the appropriate defence responses. Moreover, this research paves the way for optimal biotechnological application of natural elicitors such as OGs for sustainable crop protection.

## CONFLICT OF INTEREST

None of the major findings of the research described here has been submitted to other journals or described elsewhere.

## Supporting information


**Figure S1** Schematic model of the leaf of root treatments and nomenclature of the different samples harvested.

**Figure S2** Phytohormone levels in roots and leaves of tomato plants upon treatment with a OG solution. (a) Local responses to OG treatment in leaves. (b). Local and systemic responses to OGs in roots. (c) Systemic responses in leaves to OG application in leaves or roots.

**Figure S3** PCA of the metabolic reprograming in roots and leaves after OGs perception.

**Figure S4** Relative quantification of the alkaloid anatalline in leaves and roots upon OG treatment in the roots (6 hpt).

**Figure S5** β‐1,3‐glucanase enzymatic activity in leaves upon OGs treatment (6 hpt).

**Table S1** Primers used in this study for gene expression analysis.
