# Supplementary figures and images for: Roots drive oligogalacturonide‐induced systemic immunity in tomato
Source: Plant Cell Environ. 2020 Nov 3;44(1):275–89. doi: 10.1111/pce.13917 (PMC7883634; doi:10.1111/pce.13917)

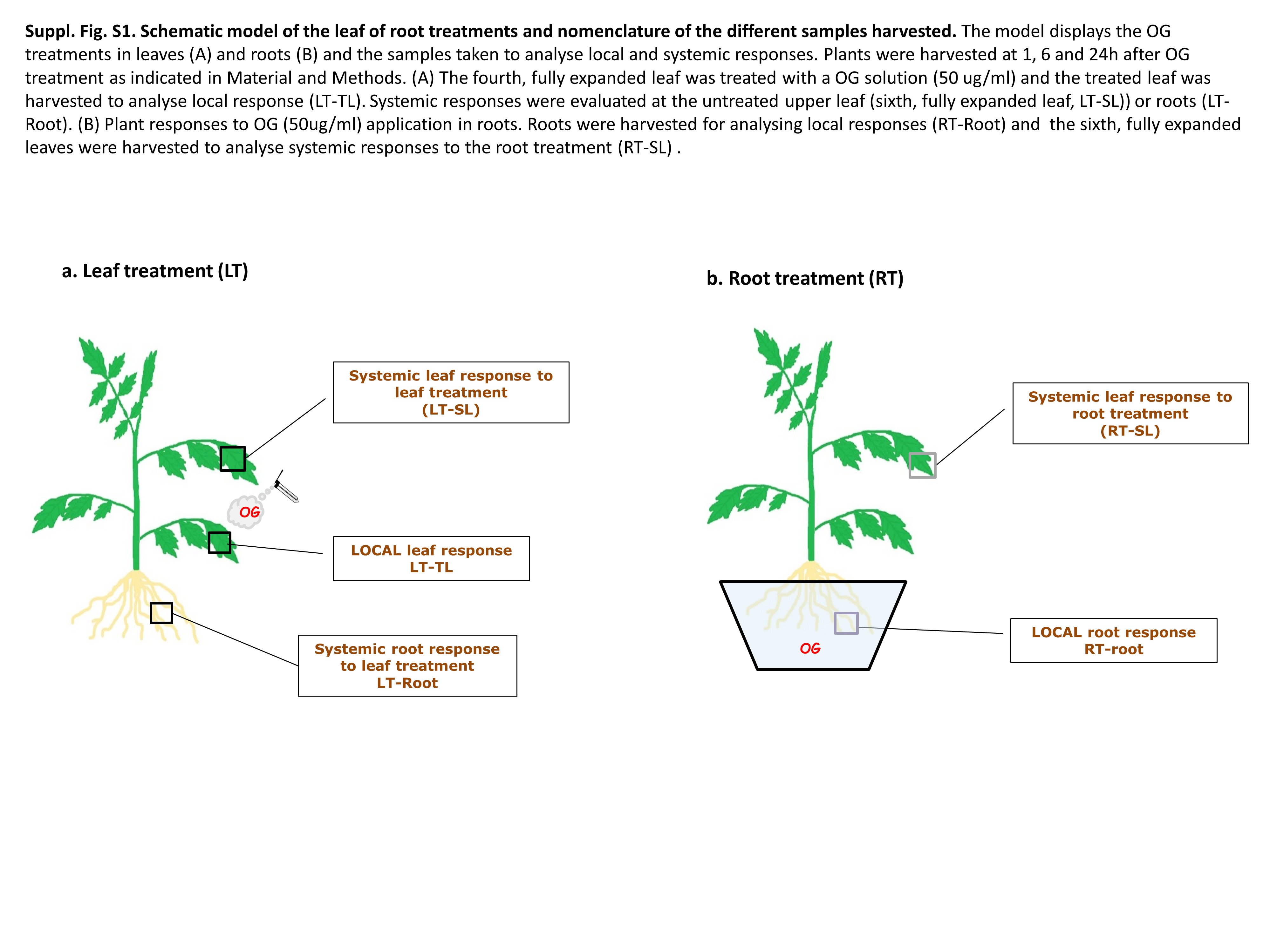

Supplement: Supplementary file 1 — Figure S1 Schematic model of the leaf of root treatments and nomenclature of the different samples harvested. [file PCE-44-275-s001.TIF]

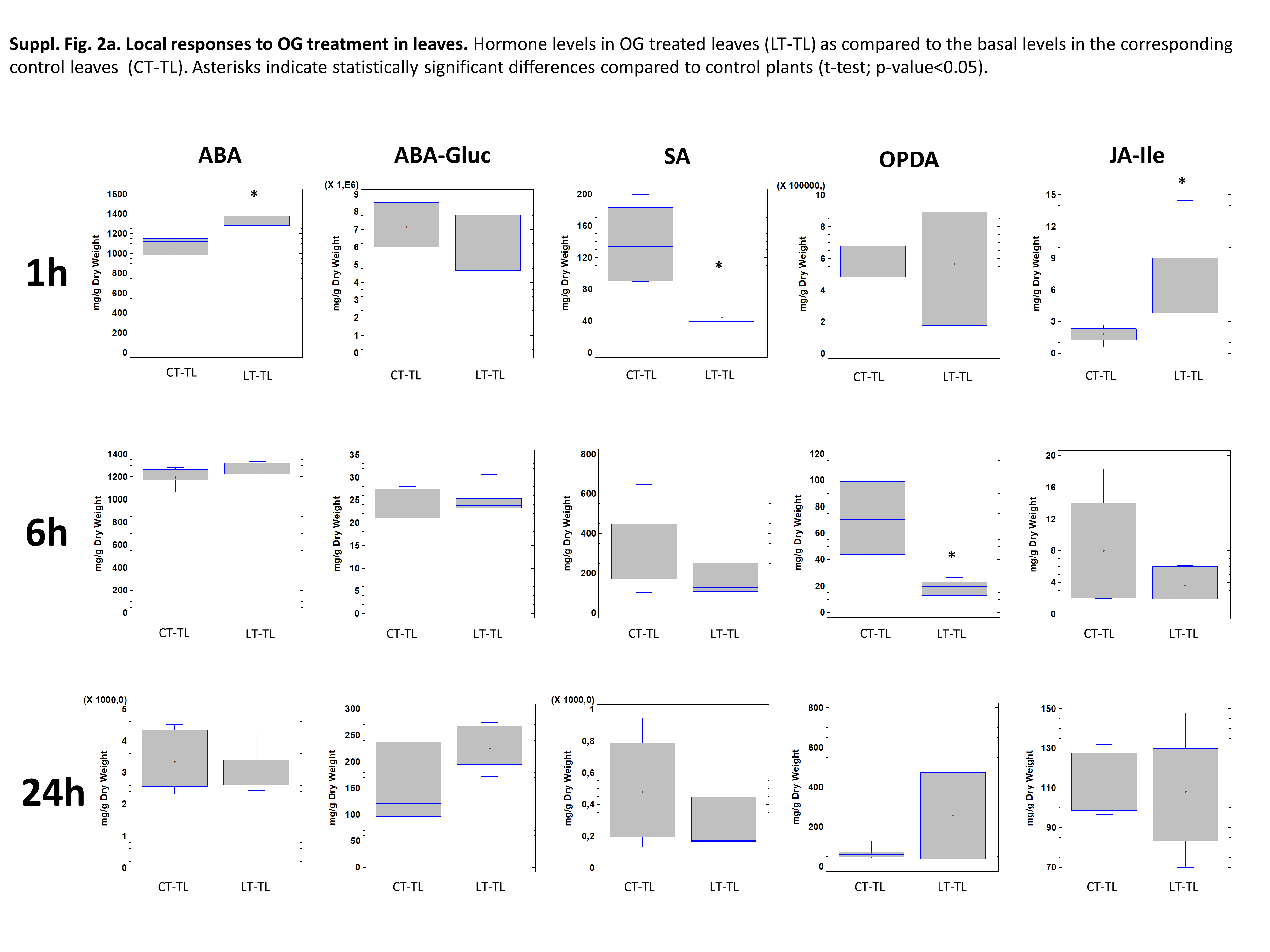

Supplement: Supplementary file 2 — Figure S2 Phytohormone levels in roots and leaves of tomato plants upon treatment with a OG solution. (a) Local responses to OG treatment in leaves. (b). Local and systemic responses to OGs in roots. (c) Systemic responses in leaves to OG application in leaves or roots. [file PCE-44-275-s002.zip › PCE_13917_Suppl Fig2a_FINAL.TIF]

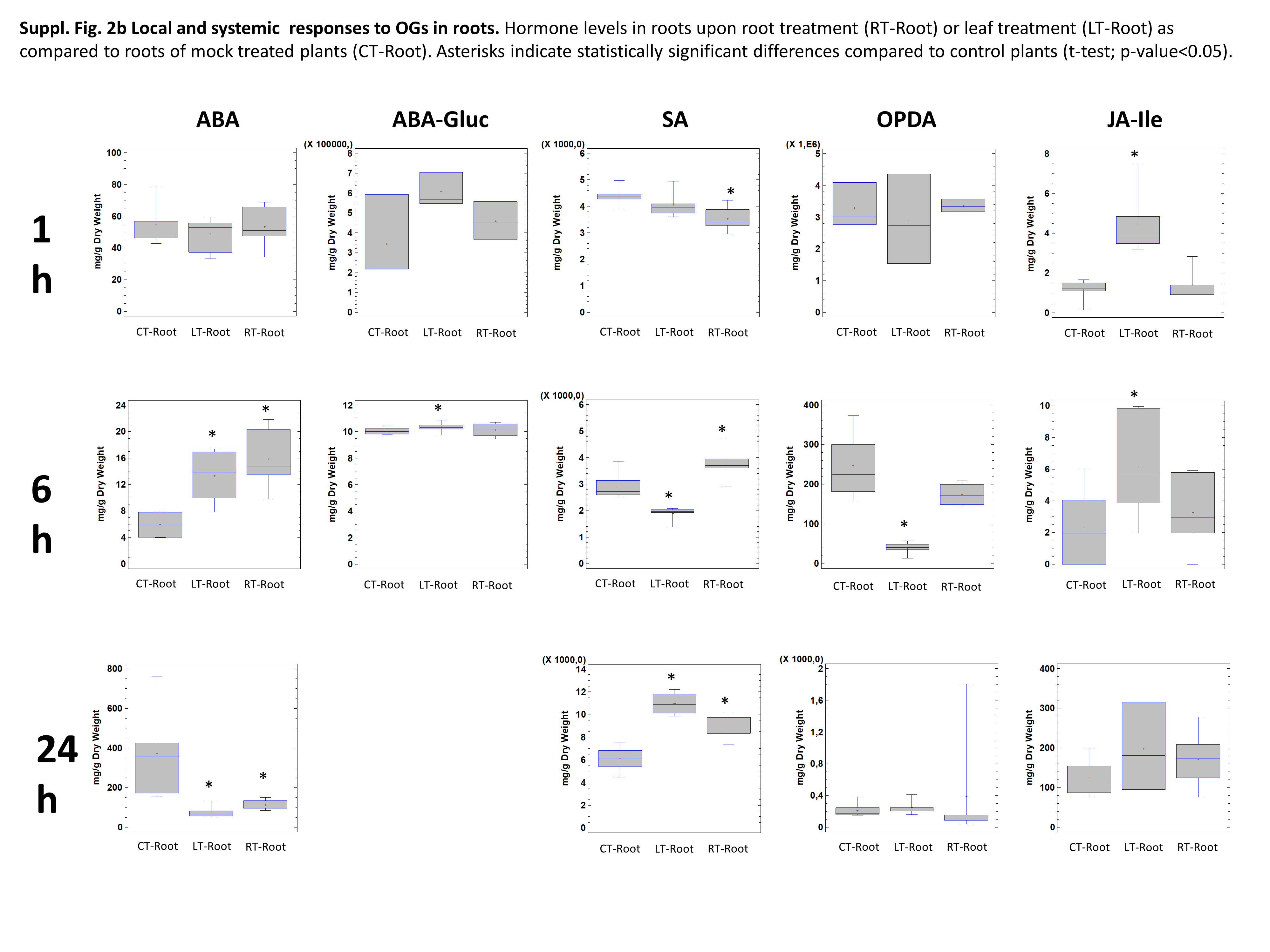

Supplement: Supplementary file 2 — Figure S2 Phytohormone levels in roots and leaves of tomato plants upon treatment with a OG solution. (a) Local responses to OG treatment in leaves. (b). Local and systemic responses to OGs in roots. (c) Systemic responses in leaves to OG application in leaves or roots. [file PCE-44-275-s002.zip › PCE_13917_Suppl Fig2b_FINAL.TIF]

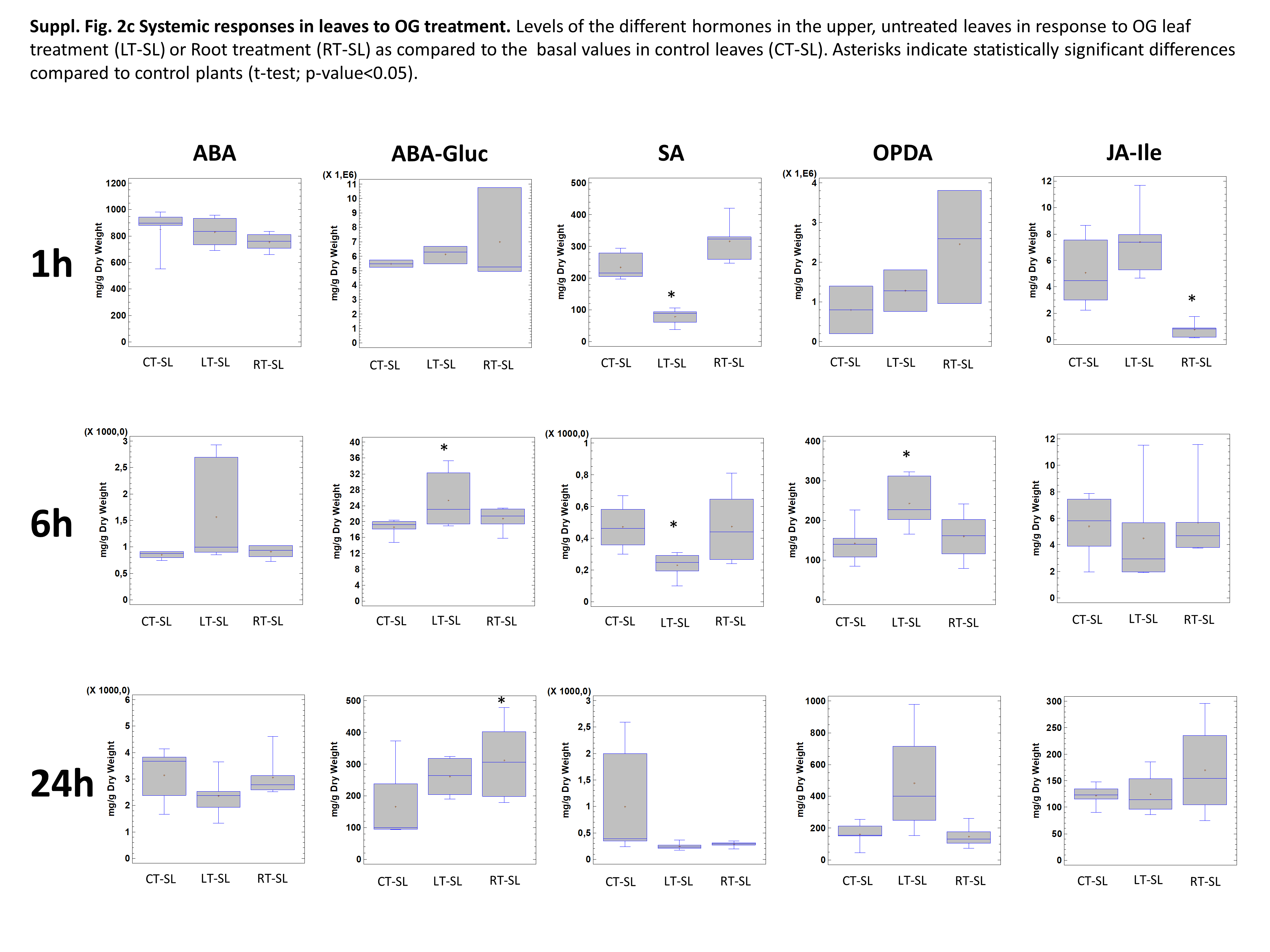

Supplement: Supplementary file 2 — Figure S2 Phytohormone levels in roots and leaves of tomato plants upon treatment with a OG solution. (a) Local responses to OG treatment in leaves. (b). Local and systemic responses to OGs in roots. (c) Systemic responses in leaves to OG application in leaves or roots. [file PCE-44-275-s002.zip › PCE_13917_Suppl Fig2c_FINAL.TIF]

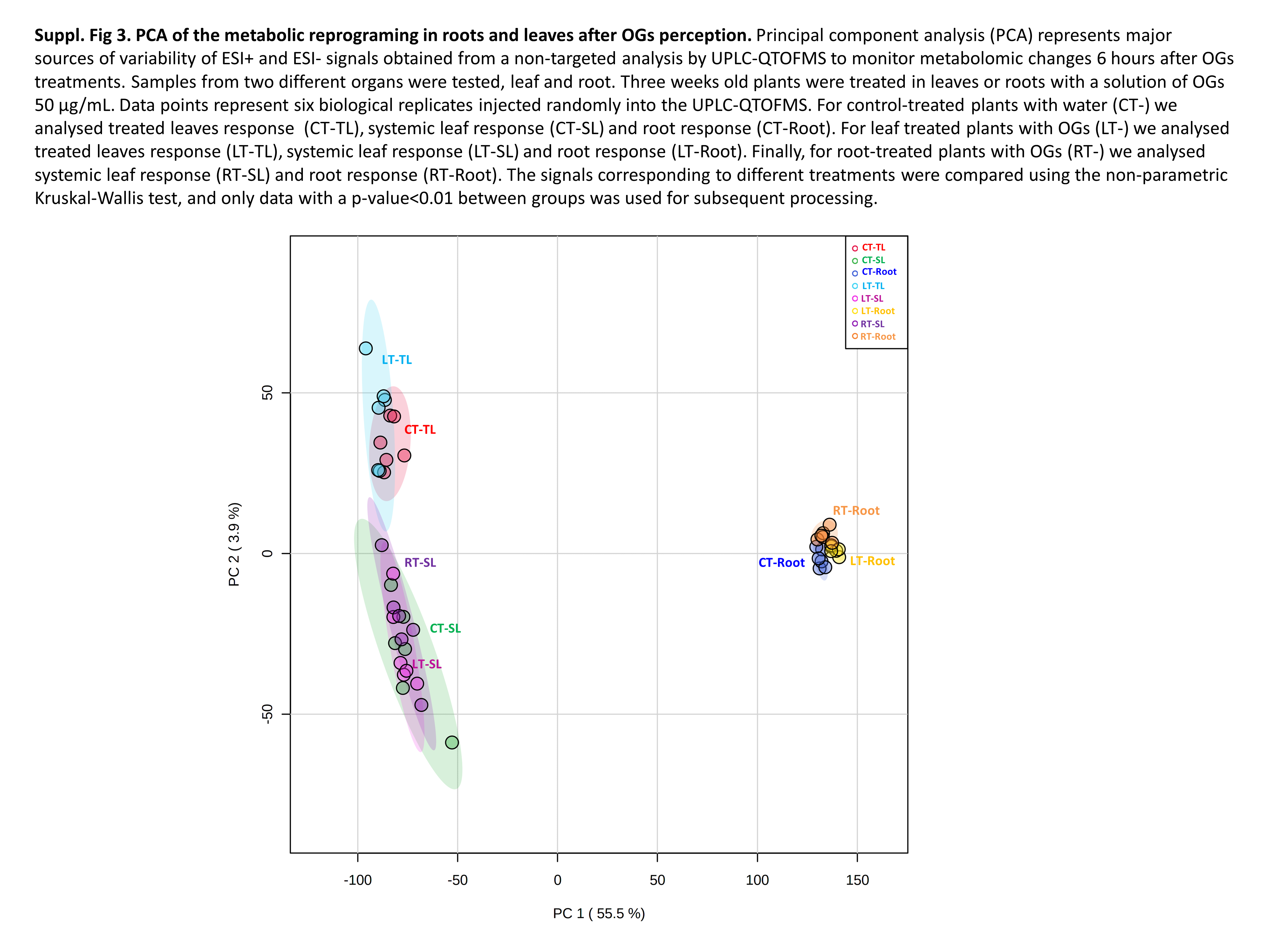

Supplement: Supplementary file 3 — Figure S3 PCA of the metabolic reprograming in roots and leaves after OGs perception. [file PCE-44-275-s003.TIF]

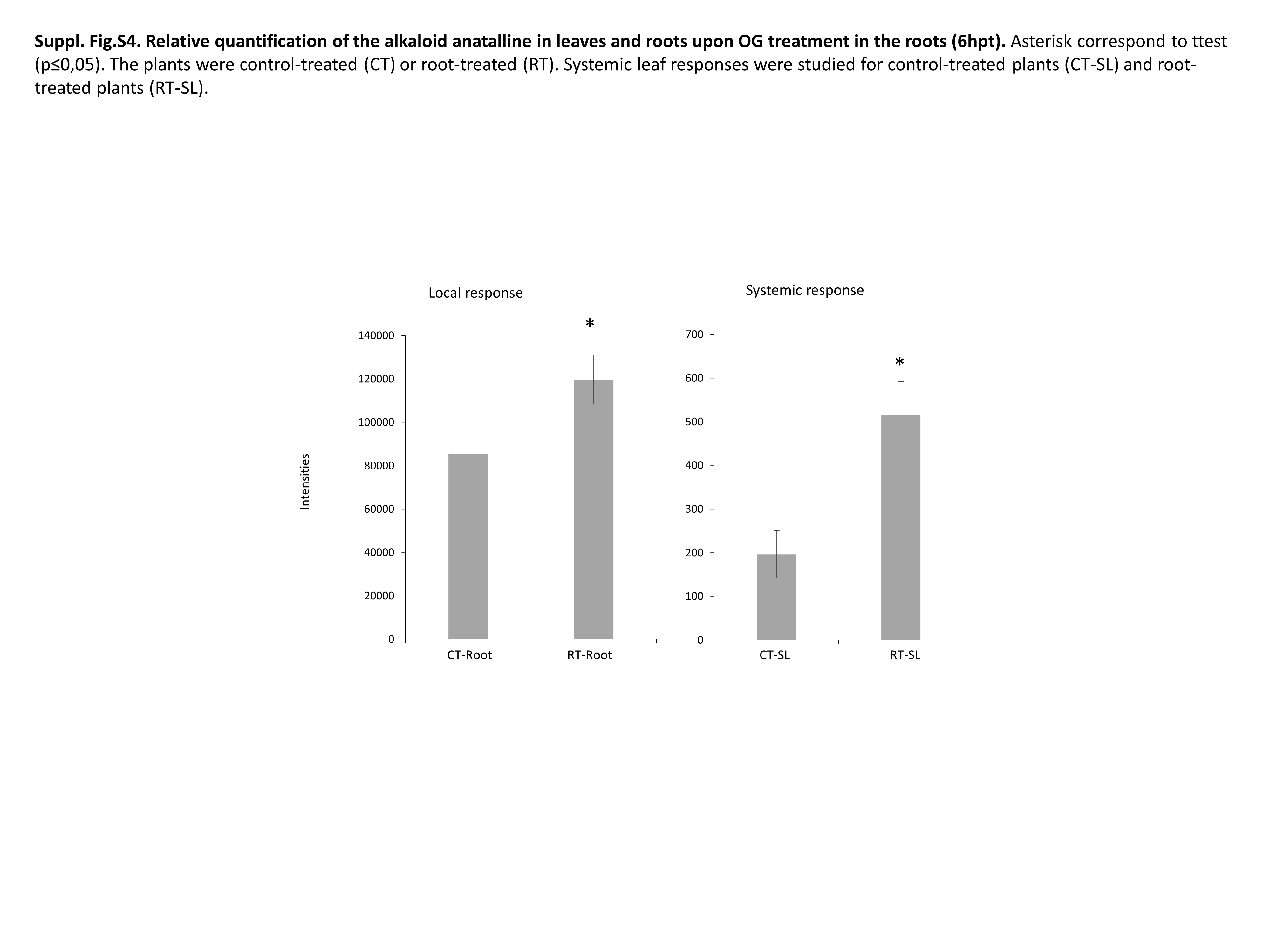

Supplement: Supplementary file 4 — Figure S4 Relative quantification of the alkaloid anatalline in leaves and roots upon OG treatment in the roots (6 hpt). [file PCE-44-275-s004.TIF]

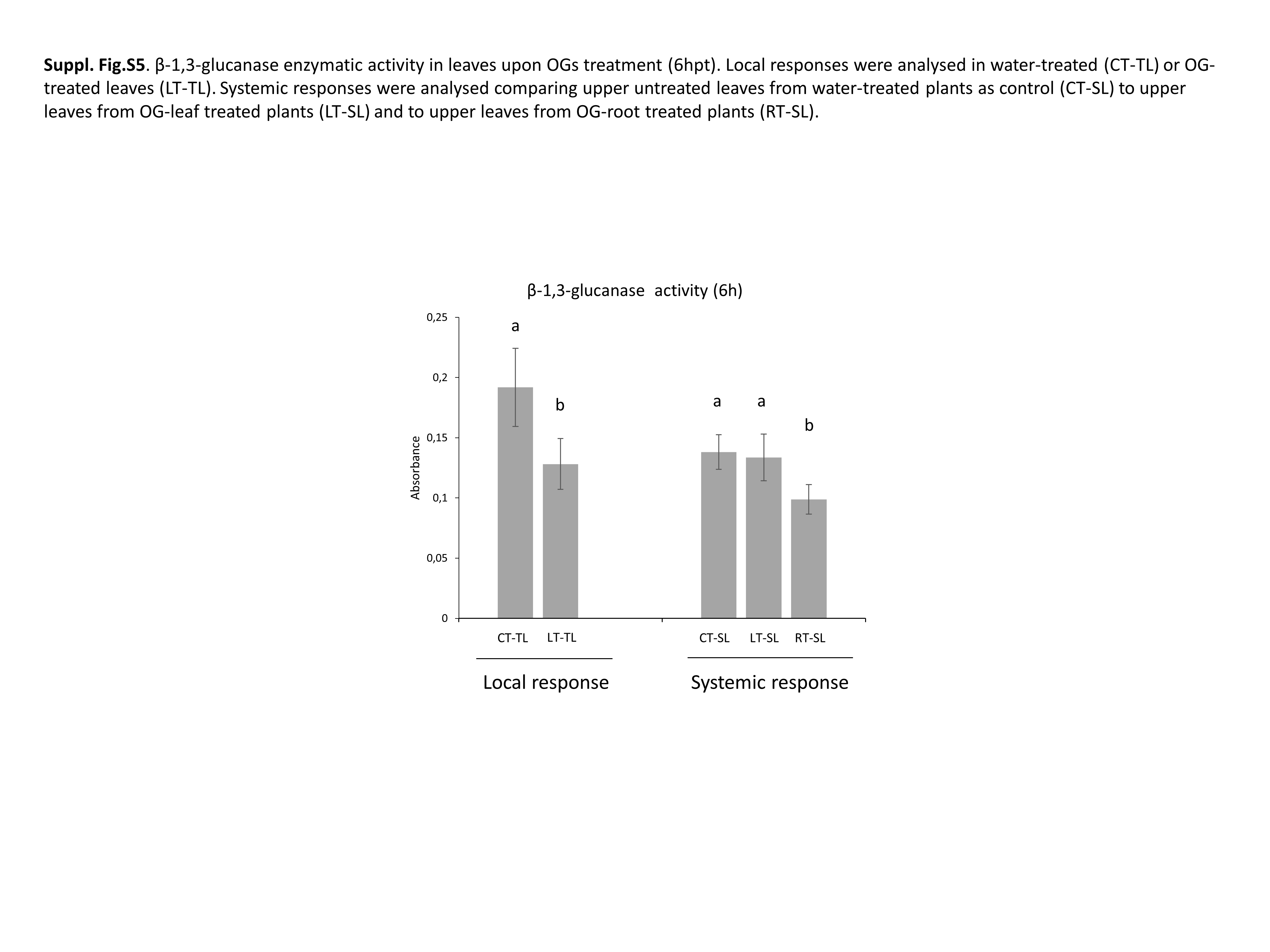

Supplement: Supplementary file 5 — Figure S5 β‐1,3‐glucanase enzymatic activity in leaves upon OGs treatment (6 hpt). [file PCE-44-275-s005.TIF]
