# Supplementary material for: Roots drive oligogalacturonide‐induced systemic immunity in tomato
Source: Plant Cell Environ. 2020 Nov 3;44(1):275–89. doi: 10.1111/pce.13917 (PMC7883634; doi:10.1111/pce.13917)
Supplement: Supplementary file 6 — Table S1 Primers used in this study for gene expression analysis. [file PCE-44-275-s006.docx]

**Table S1.** Primers used in this study for gene expression analysis.

| **ID** | **Gene** | **Primers (5`-3`)** |
| --- | --- | --- |
| CN384809 | 1-aminocyclopropane-1-carboxylic acid oxidase 1 (*ACO1*) ^1^ | GGACTCCGCGCTCATACAGA  ATAGAGTGGCGCATGGG |
| U37840 | Lipoxygenase D (*LOXD*) ^1^ | GACTGGTCCAAGTTCACGATCC  ATGTGCTGCCAATATAAATGGTTCC |
| Z97215 | 9-cis-epoxycarotenoid (*NCED*) ^1^ | ACCCACGAGTCCAGATTTC  GGTTCAAAAAGAGGGTTAGC |
| NM001247876 | β-1,3-glucanase (*Glub*) ^2^ | CCATCACAGGGTTCATTTAGG  CCATCCACTCTCTGACACAACT |
| M83314 | Phenylalanin ammonia lyase (*PAL*) ^2^ | CGTTATGCTCTCCGAACATC  GAAGTTGCCACCATGTAAGG |
| Solyc09g091510 | Chalcone synthase 1 (*CHS1*.1) ^3^ | GTTCCGTGGACCCAGTGAAT  AAAAGGGCTTGGCCTACCA |
| Solyc05g010320 | Chalcone isomerase 1 (*CHI1*.1) ^3^ | GAAGCAGTGCTGGATTCCATAAT  GTTTTTCACAAACCAACAGTTCTGAT |
| Solyc06g053510 | putrescine N-methyltransferase (*PMT*) ^6^ | CACTTGGTTCCATTCCATCC  TGAGCAGCTTTCACAAATGC |
| Solyc03g078400 | SlActin2 ^5^ | TTGCTGACCGTATGAGCAAG  GGACAATGGATGGACCAGAC |
| Solyc06g005060 | Elongation factor 1-α *(SlEF*-1α) | GATTGGTGGTATTGGAACTGTC  AGCTTCGTGGTGCATCTC |
| Solyc04g081490 | Β-tubulin *(SLTUB)* | TGGGCTGAAGATGGCATCCACG  GCCTTGCGCCTGAACACATGGC |

**^1^López-Ráez JA, Charnikhova T, Fernández I, Bouwmeester H, Pozo MJ**. 2010. Arbuscular mycorrhizal symbiosis decreases strigolactone production in tomato. Journal of Plant Physiology **168**, 294–297.

**^2^Martinez-Medina A, Fernandez I, Sánchez-Guzmán M, Jung S, Pascual J, Pozo MJ**. 2013. Deciphering the hormonal signalling network behind the systemic resistance induced by Trichoderma harzianum in tomato. Frontiers in Plant Science, **4.**

**^3^Ballester AR, Tikunov Y, Molthoff J, Grandillo S, Viquez-Zamora M, de Vos R, de Maagd RA., van Heusden S, Bovy AG**. 2016. Identification of Loci Affecting Accumulation of Secondary Metabolites in Tomato Fruit of a Solanum lycopersicum × Solanum chmielewskii Introgression Line Population. Frontiers in Plant Science, **7.**

**^4^M. Teuber, M.E. Azemi, F. Namjoyan, A.-C. Meier, A. Wodak, W. Brandt, B. Dräger.** 2007. Putrescine N-methyltransferases – a structure–function analysis. Plant Mol. Biol **63**, 787-801

**^5^Yan L, Zhai Q, Wei J, Li S, Wang B, Huang T, Du M, Sun J, Kang L, Li CB, Li C.** 2013. Role of Tomato Lipoxygenase D in Wound-Induced Jasmonate Biosynthesis and Plant Immunity to Insect Herbivores. PLoS Genet **9**, e1003964.
